# Characterization of spherical domains at the polystyrene thin film–water interface

**DOI:** 10.3762/bjnano.7.51

**Published:** 2016-04-20

**Authors:** Khurshid Ahmad, Xuezeng Zhao, Yunlu Pan, Danish Hussain

**Affiliations:** 1 Key Laboratory of Micro-Systems and Micro-Structures Manufacturing, Ministry of Education and School of Mechatronics Engineering, Harbin Institute of Technology, Harbin 150001, P.R. China; 2 Department of Mechanical Engineering, Main Campus, University of Engineering and Technology, Peshawar, Pakistan; 3 State Key Laboratory of Robotics and Systems and School of Mechatronics Engineering, Harbin Institute of Technology, Harbin 150001, P.R. China

**Keywords:** AFM, blisters, contaminants, defects, nanobubbles, water permeation

## Abstract

Spherical domains that readily form at the polystyrene (PS)–water interface were studied and characterized using atomic force microscopy (AFM). The study showed that these domains have similar characteristics to micro- and nanobubbles, such as a spherical shape, smaller contact angle, low line tension, and they exhibit phase contrast and the coalescence phenomenon. However, their insensitivity to lateral force, absence of long-range hydrophobic attraction, and the presence of possible contaminants and scratches on these domains suggested that these objects are most likely blisters formed by the stretched PS film. Furthermore, the analysis of the PS film before and after contact with water suggested that the film stretches and deforms after being exposed to water. The permeation of water at the PS–silicon interface, caused by osmosis or defects present on the film, can be a reasonable explanation for the nucleation of these spherical domains.

## Introduction

Thin films of several nanometer thickness have long been a topic of interest for researchers. The application of such thin films has been demonstrated in nonvolatile memory devices [1], sensors [2–3], for the modification of emissive properties of glass [4–5], and for the modification of surface properties [6–8] (e.g., hydrophobicity, oleophobicity). The study of the thermal [4], optical [5], mechanical [9–10], and interfacial [6–8] properties of thin films is a broader area of interest. Various physical and chemical processes have been used to produce such films. Polystyrene (PS) is one of the most widely used materials for the preparation of thin films. Thin PS films have been prepared by spin coating [11–15]. So far, these films have been used in different studies related to surface and interface science, for example, to study boundary slip and micro-/nanobubble formation [15–19]. Nanobubbles are gaseous domains that may be found at a solid–liquid interface. Over the past few decades, dedicated research has been carried out on nanobubbles at the solid–liquid interface. AFM has been proven to be a promising technique for the imaging and analysis of micro-/nanobubbles. Studies have reported surface micro-/nanobubbles on thin PS films immersed in water [15–22]. Bubbles ranging from tens of nanometers to several micrometers have been reported on thin PS films [15–19,21], and the different characteristics of bubbles have been studied by various research groups [15–16,19,23–24]. Studies have shown that the contact angle of the nanoscale gaseous bubbles is smaller than the macroscopic gaseous bubbles (measured from the air side) [15]. It has also been reported that the contact angle of the gaseous bubble increases with the lateral size in each independent size scale [15]. Similarly, increased surface roughness and the influx of gas from the interfacial gas enrichment favors formation of larger gaseous bubbles [15,19]. In addition, other studies have reported different phenomena on the PS-coated surface such as dewetting from the silicon surface and formation of nanoindents and blisters [11,25–28]. It has been shown that the PS film can be dewetted from the silicon surface upon the contact with water [11]. Wang et al. [25] found that the nanobubbles can form nanoindents on the PS-coated surface due to high gas pressure inside the bubble. Additionally, Maebayashi [12] and Berkelaar et al. [26] reported on the nucleation process of blisters on PS films. These studies reveal the possibility that nanobubbles may coexist with blisters on thin PS films. This study employs AFM and optical microscopy to characterize the spherical-shaped domains that readily nucleate on the PS film after immersion in DI water. The radius, height, contact angle (CA) and line tension are analyzed in detail. The coalescence, stiffness and phase contrast analysis were also studied. Moreover, changes in surface topography, before and after the contact with water, have also been discussed.

## Experimental

### Materials and equipment

The following materials and equipment were used in this study: deionized water purified with a Milli-Q A10 system, silicon dioxide (Lijing, LLC, China), polystyrene beads (average MW ≈350,000; Aldrich, USA), AFM cantilevers (RTESPA, DNP, SNL; Bruker, USA), AFM (Innova; Bruker, USA), digital microscope (KH1300; Hirox, Japan), spin coater (KW-4A; SETCAS Electronics Co. Ltd., Beijing, China), and drop meter (MAIST, Vision, China).

#### Sample preparation

**Figure 1:**
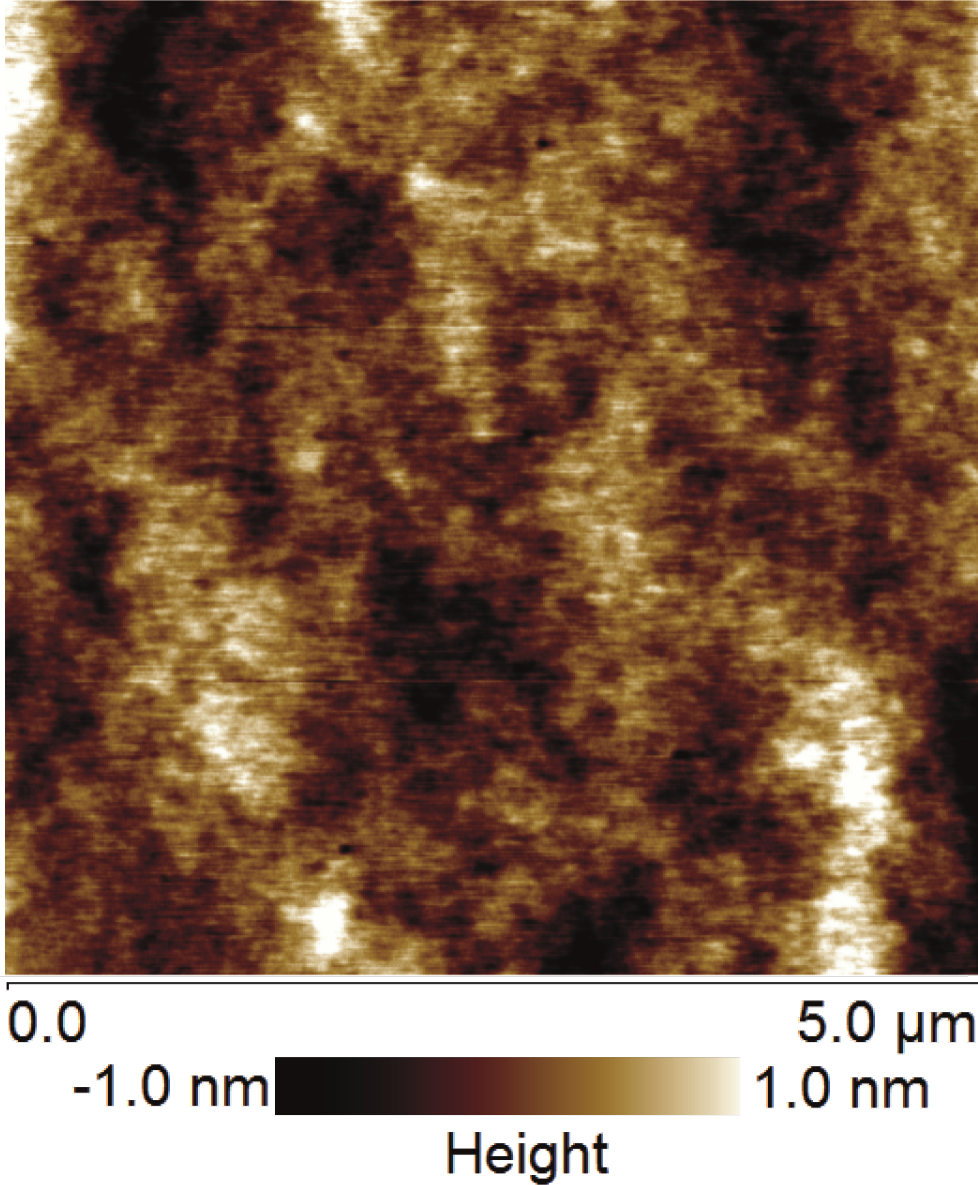
Topographic image of a typical PS-coated surface. The roughness average (*R*_a_) was 0.27 nm, the root mean square (RMS) was 0.35 nm, and the maximum peak-to-valley height of the image (*R*_max_) was 2.86 nm.

Polystyrene thin films were spin-coated onto silicon dioxide wafers. Prior to spin coated, the silicon dioxide wafers were cleaned using piranha solution of 3:1 (v/v) sulfuric acid/hydrogen peroxide for 30 min [8]. The wafers were further cleaned with acetone, ethanol and DI water in an ultrasonic sonicator, followed by drying with clean, compressed air. The PS solution (10:1 v/w of toluene/PS) was prepared by dissolving PS beads in toluene. The PS film was spin-coated each of the silicon dioxide wafers at a speed of 3000 rpm. Afterward, the PS-coated surfaces were cured in an electric oven for 4 h at 50 °C. The surfaces were rinsed with DI water and dried with clean, compressed air. In certain cases, the surfaces were not rinsed before scanning with AFM. The CA of the water on the PS-coated surfaces was 85 ± 4°. The surfaces were scanned in air using tapping mode atomic force microscopy (TM-AFM). A typical image of the PS-coated surface is given in Figure 1.

#### Atomic force microscopy analysis

The TM-AFM technique was used to analyze the PS-coated surfaces and the spherical domains. A set point ratio of 85 ± 5% of the free air amplitude was used for scanning in the liquid. We used silicon nitride cantilevers with a nominal tip radius of 20 nm and nominal stiffness of 0.05 N/m. The resonance frequency of the cantilever immersed in DI water was 35.0 kHz. Furthermore, an average scan rate of 1 Hz was used to image the surface topography and the micro/nano spherical domains. Moreover, the thickness of the PS film, measured with AFM using the scratch profile method, was 42.0 ± 7 nm.

## Results and Discussion

### Analysis of radius, lateral size and height of spherical objects on PS thin films

**Figure 2:**
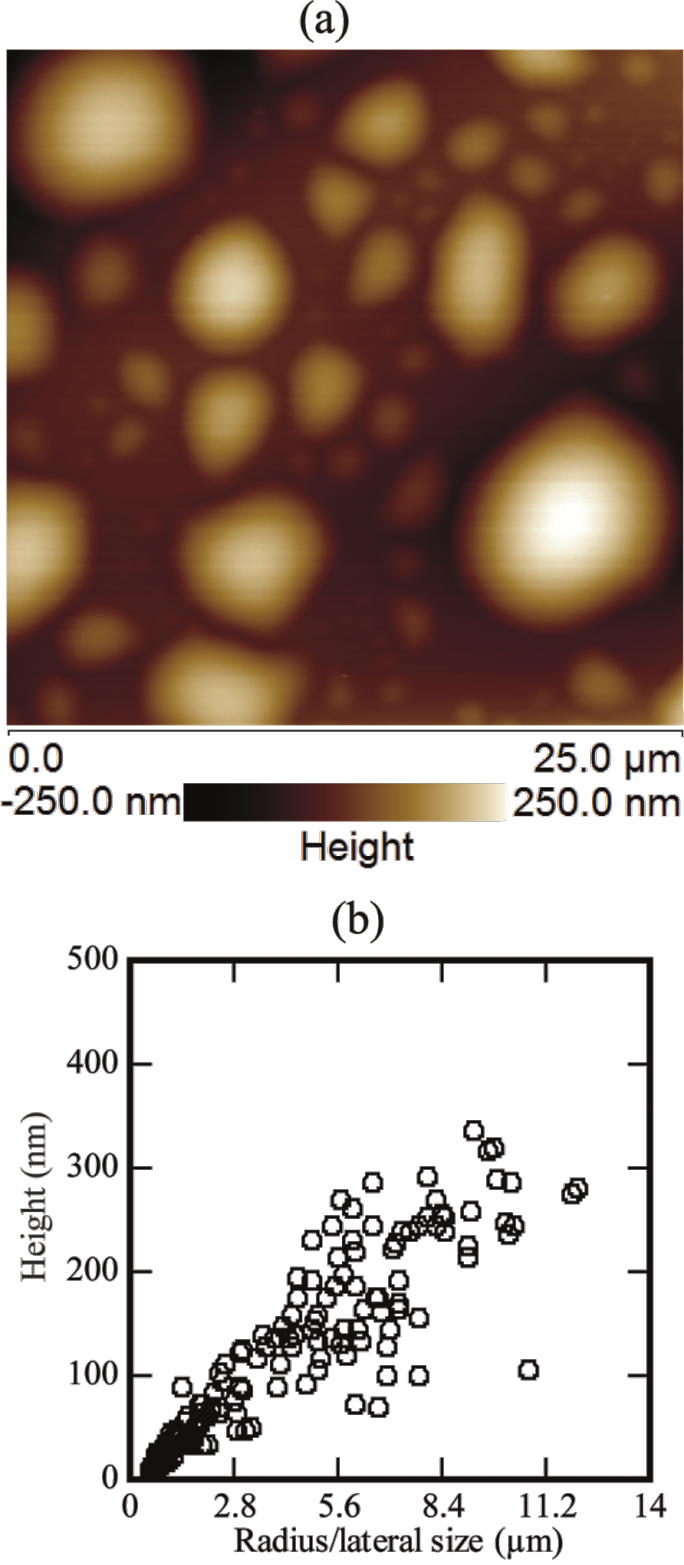
(a) AFM image of micro/nano spherical domains on a PS-coated surface, immersed in DI water. A number of spherical or nearly spherical objects can be seen in the image. (b) Summary of the height and radius/lateral size of the spherical domains. Additional images of the so-called spherical domains are shown in Supporting Information File 1, Figure S1.

Various-sized spherical or nearly spherical objects were found at the PS–water interface. The size ranged from several hundreds of nanometers to several micrometers, as shown in Figure 2a. The dimensions of the spherical objects were obtained by making the tip correction [15,29]. The apparent radius of curvature (*R*_ac_), corrected radius of curvature (*R*_c_), radius of the bubble (*R*_b_), and contact angle (θ) were calculated using the following equations [15,18]:

[1]
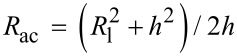


[2]
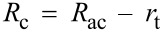


[3]
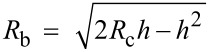


[4]
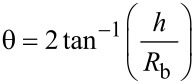


where, *R*_1_ is the lateral size/radius of the domain without tip correction, *h* is the height of the spherical domain, and *r*_t_ is the nominal radius of the tip. A number of spherical domains were found on different PS-coated surfaces immersed in DI water. A typical image showing a number of spherical or nearly spherical domains is shown in Figure 2a. These domains were analyzed through the Nanoscope analysis software (Bruker, USA). The details of the radius/lateral size and the height were collected. A brief summary is given in Figure 2b. Figure 2b illustrates that the height of the spherical domains changes almost linearly with respect to the radius/lateral size. The lateral size varied between 0.5 and 14.0 µm while the height varied from 7.0 to 300.0 nm.

### Analysis of the contact angle

**Figure 3:**
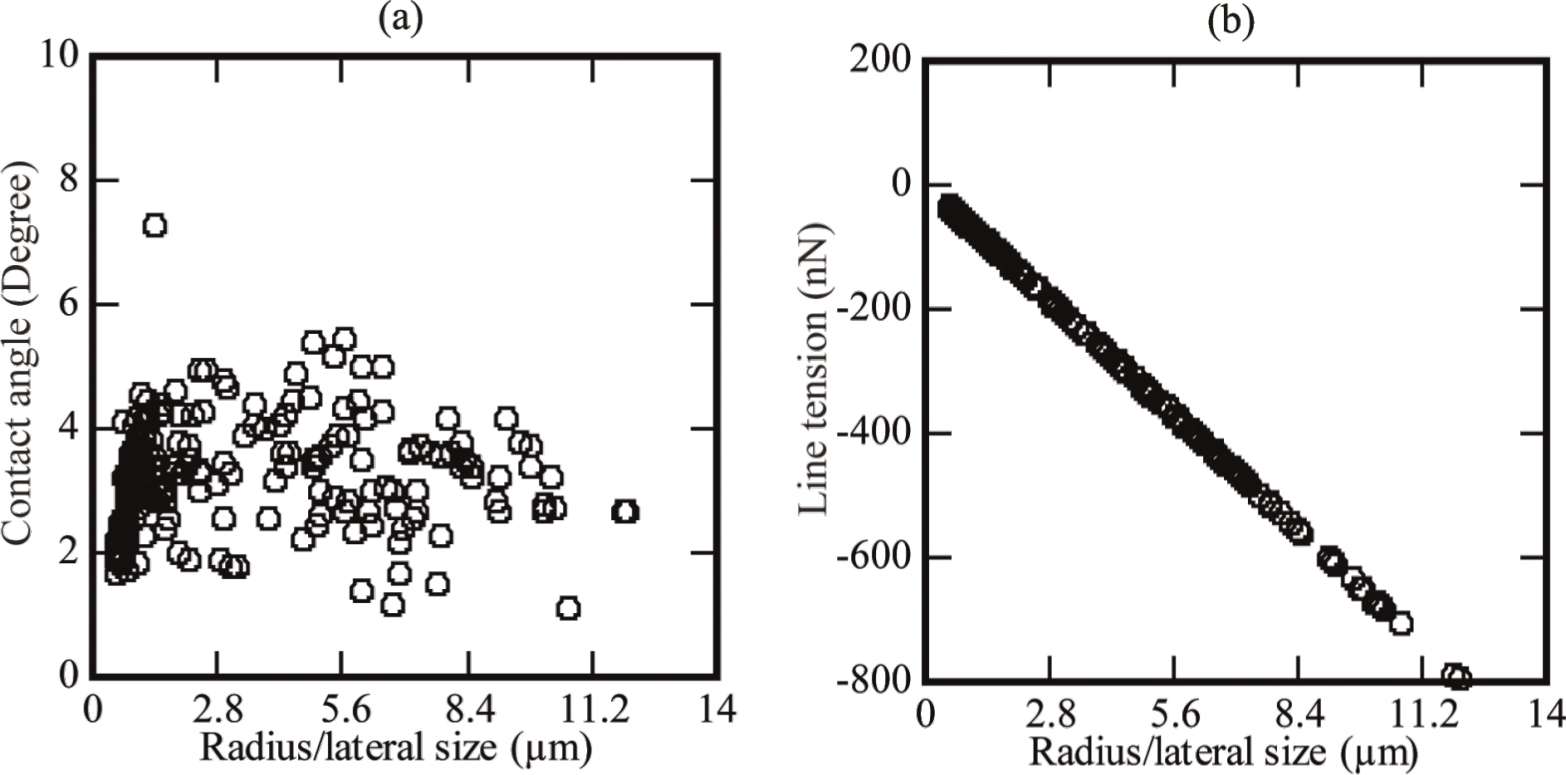
(a) Summary of the contact angle versus the radius/lateral size of the spherical objects. (b) Summary of the line tension of the spherical objects on PS-coated surfaces.

The contact angle of these domains was also analyzed. The analysis showed a very small contact angle when measuring from the inner side of the spherical domain. Generally, the contact angles were approximately in the range of 2.0 to 6.0°. A summary of the contact angles of the spherical domains found on various PS-coated surfaces immersed in DI water is given in Figure 3a. Figure 3a shows that the contact angle changes linearly with respect to radius/lateral size up to 2.0 µm. Beyond this size, the contact angle changes in such a way that an exact correlation becomes difficult.

### Analysis of line tension

The line tension is defined as the excess energy per unit length of the three phase contact line [30]. The magnitude of the line tension affects the shape of the bubble [29,31–32]. Negative as well as positive values of line tension have been reported in previous studies [32–34]. Therefore, the sign of the line tension is still a controversial debate [35]. Studies have shown that the magnitude of line tension for nanobubbles ranges from nN to pN [32–35]. The line tension of the spherical domains analyzed in this study was calculated using the following equation [19,33,35]:

[5]
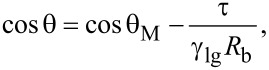


where θ is the contact angle of the spherical domains, θ_M_ is the macroscopic contact angle, τ is the line tension, and γ_lg_ is the surface tension. The results were plotted against the radius of the spherical objects, as shown in Figure 3b. It was found that the line tension varies linearly with the radius/lateral size. This analysis also showed that the magnitude of the line tension of these objects is several orders of magnitude larger (30 nN to 800 nN) than some of the previously reported values of the line tension for nanobubbles [19,33,35]. However, the magnitude is still within the range proposed in other studies (10 pN to 10 µN) [35].

### Coalescence of spherical objects

**Figure 4:**
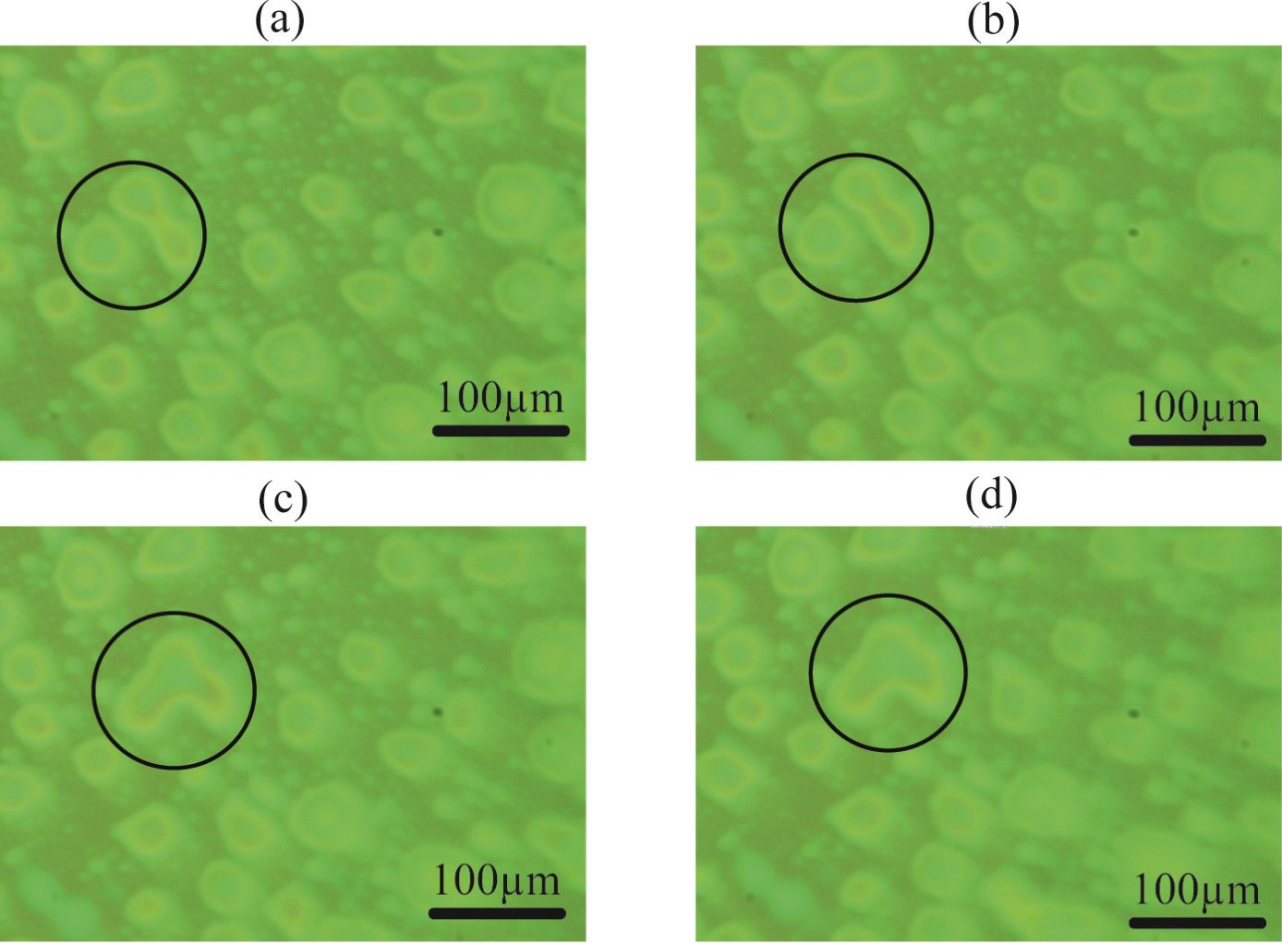
Optical images of the coalescence of spherical domains formed on PS-coated surfaces. (a–d) Time lapse images where the encircled area shows the coalescence of an irregular domain with an approximately spherical domain in the vicinity. Additional examples of coalescence are given in Supporting Information File 1, Figure S2 and Figure S3.

Based on the information collected through AFM, no coalesce was observed. However, coalescence of the objects was observed in optical microscopy (Digital Microscope, KH1300; Hirox, Japan; resolution ≈0.07 µm) experiments. Some examples of the micro-/nano spherical domains are shown in Figure 4a–d.

The study suggested that the coalescence might result in spherical and irregular-shaped domains (further information is given in Supporting Information File 1, Figure S2 and Figure S3). The presence of additional domains in the vicinity of the domains merging together can help in growth of these domains. Moreover, the shape of these domains can be affected by the presence of additional domains as well as binding of the PS film to the silicon substrate. The presence of other domains in the surrounding and less tightly bound film might favor coalescence and hence produce larger domains (see Figure 4 above, and Supporting Information File 1, Figure S2 and Figure S3). As pointed out earlier, it is also possible that the coalescence of these domains result in irregular-shaped domains, as shown in Figure 2a. Moreover, the AFM study showed that domains with lateral size ≤1.0 µm (approximately), have an almost spherical shape (see Supporting Information File 1, Figure S1).

### Stiffness of the spherical objects

**Figure 5:**
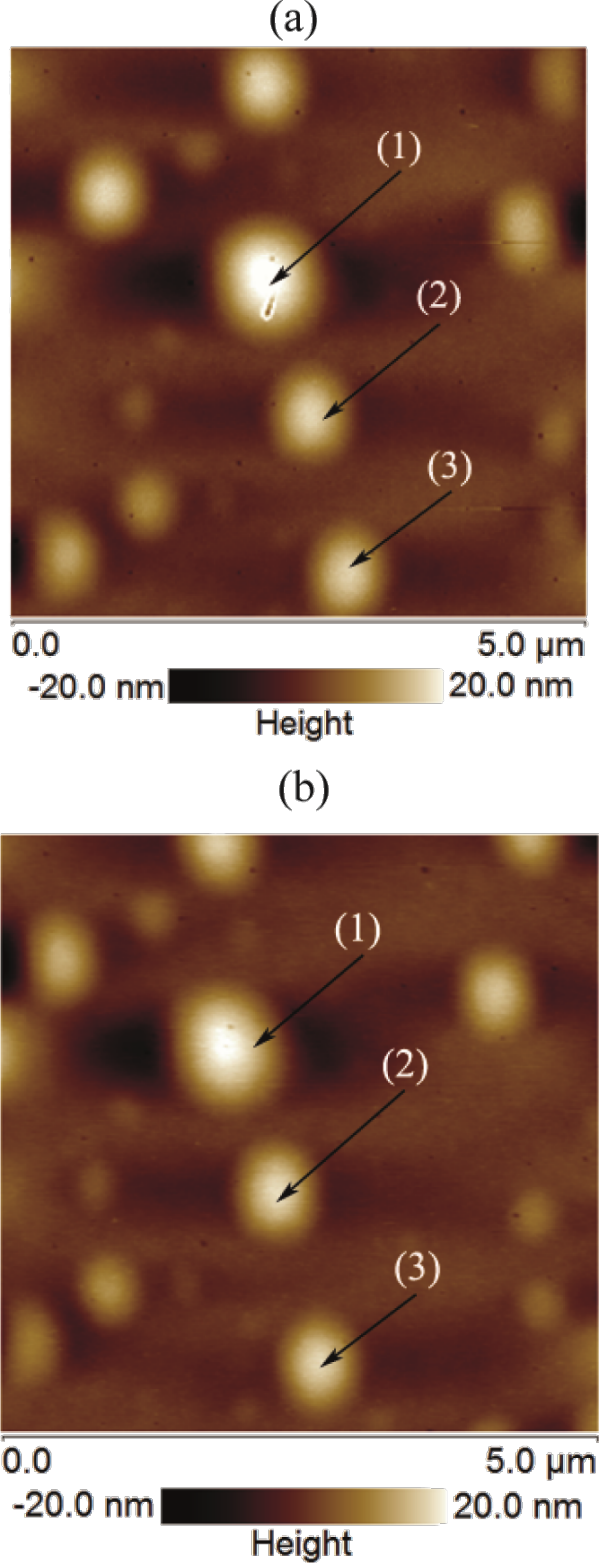
Height images of spherical objects on PS-coated surfaces obtained using (a) contact mode (b) tapping mode at 90% set point ratio.

**Figure 6:**
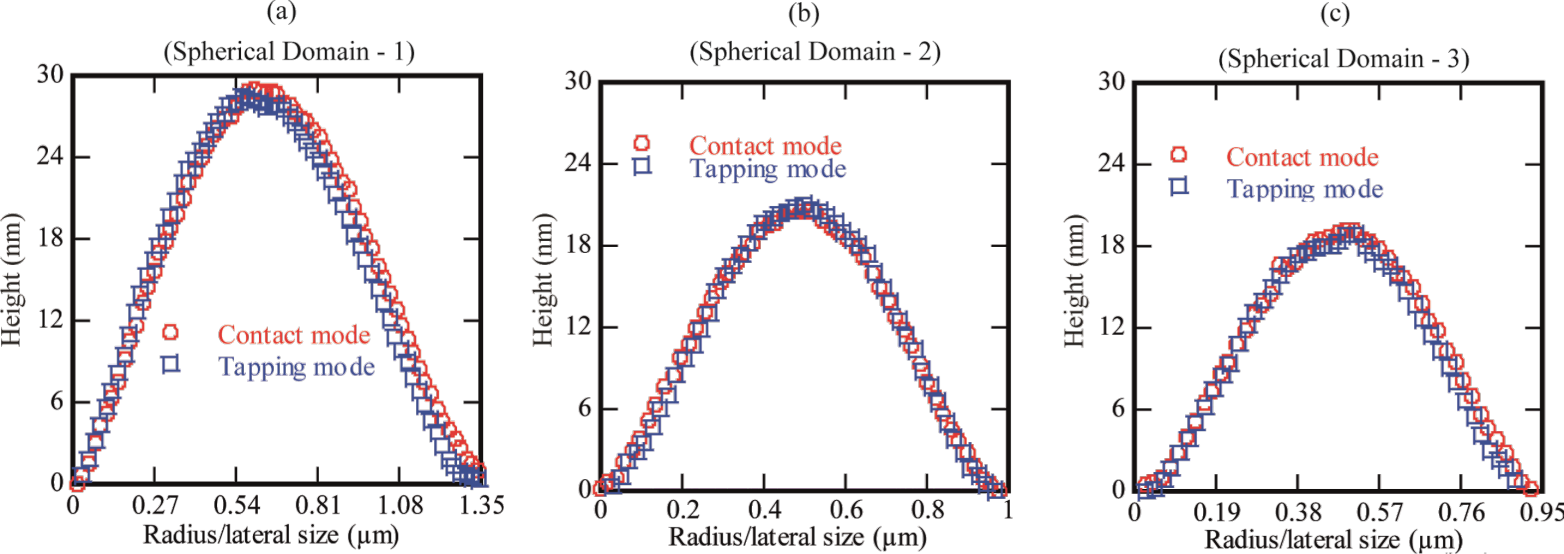
Section analysis of spherical domains imaged in contact mode and tapping mode (shown in Figure 5). (a) Section of spherical domain (1). (b) Section of spherical domain (2). (c) Section of spherical domain (3). The circles show the section of the spherical domain imaged in contact mode while the squares show the section of the spherical domains imaged in tapping mode.

**Figure 7:**
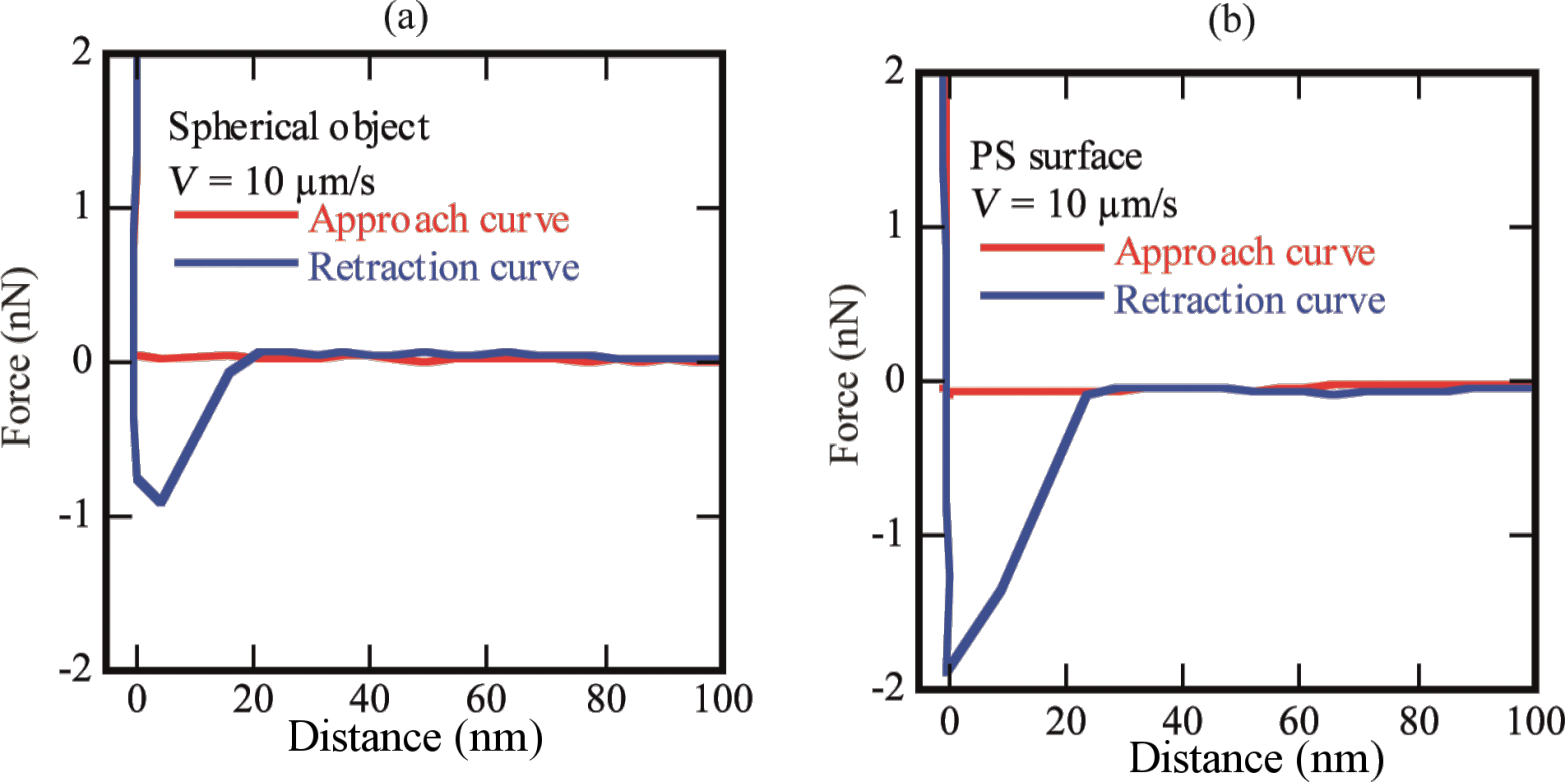
(a) Approach and retraction curves for a spherical object. (b) Approach and retraction curves for a PS-coated surface.

Nanobubbles are softer and the tip–bubble interaction can affect the shape and movement of the bubbles [19,29,36–38]. In order to differentiate the spherical domains from micro-/nanobubbles, the surface was scanned in contact mode AFM (CM-AFM) as well as TM-AFM. The typical images are shown in Figure 5. The section analysis of the spherical domains shown in the Figure 5 is given in Figure 6a–c. In Figure 6a–c there is no evident difference in the shape of the spherical domains scanned with TM-AFM or CM-AFM. It shows that these domains are stiff enough to resist the deformation due to lateral force applied by the tip in CM-AFM. Moreover, in addition to the softer nature, previous studies have also reported that the presence of gaseous bubbles give rise to a long range hydrophobic attraction force [39–41]. Therefore, to analyze this aspect, the approach and retraction force curves were obtained on a bubble as well as the PS-coated surface. The results are shown in Figure 7a,b where it is obvious that there is no clear difference in the approach force curves obtained on the spherical domain or the PS-coated surface. The so-called hydrophobic attraction force was expected in the case of the gaseous bubbles. However, these force curves are identical and suggest that the nature of these objects is not similar to that of bubbles. Moreover, additional force curves obtained on different sized spherical domains are given in Supporting Information File 1, Figure S6.

### Analysis of phase contrast and presence of contaminants on the spherical domains

**Figure 8:**
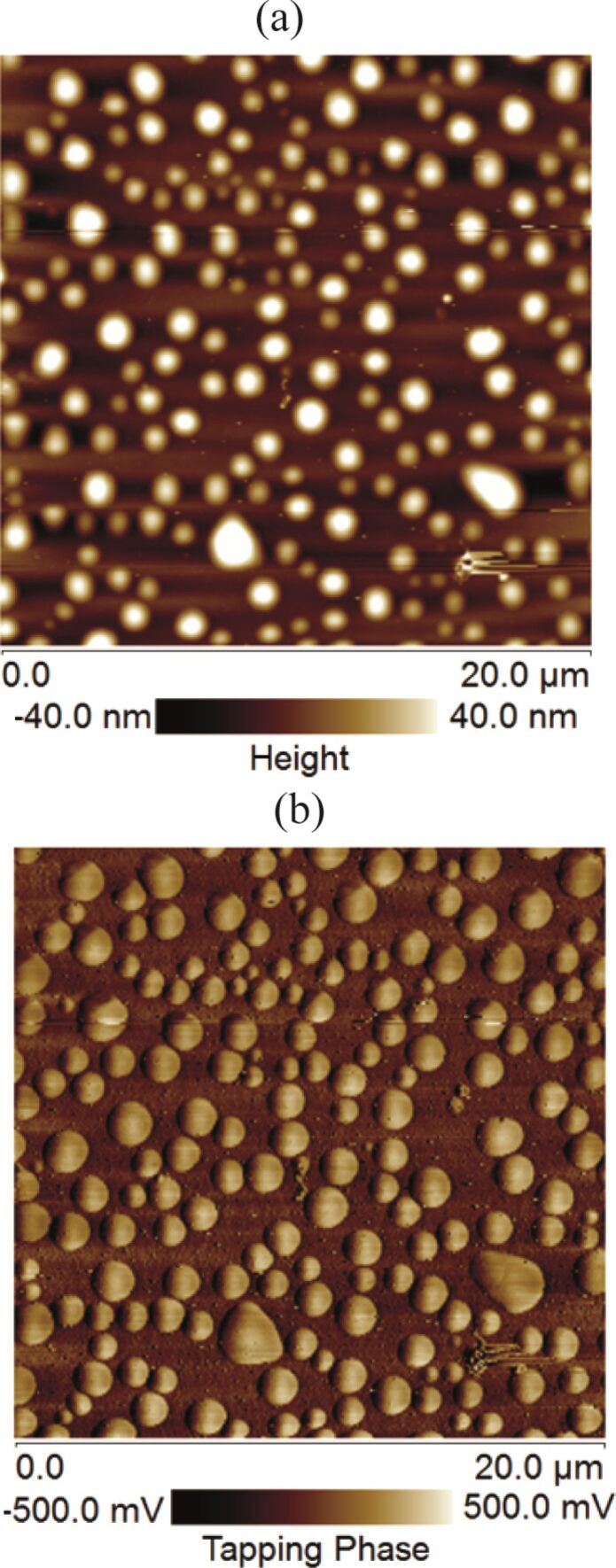
(a) Height image of spherical domains. (b) Phase image of spherical domains. A clear phase contrast can be observed in the image.

**Figure 9:**
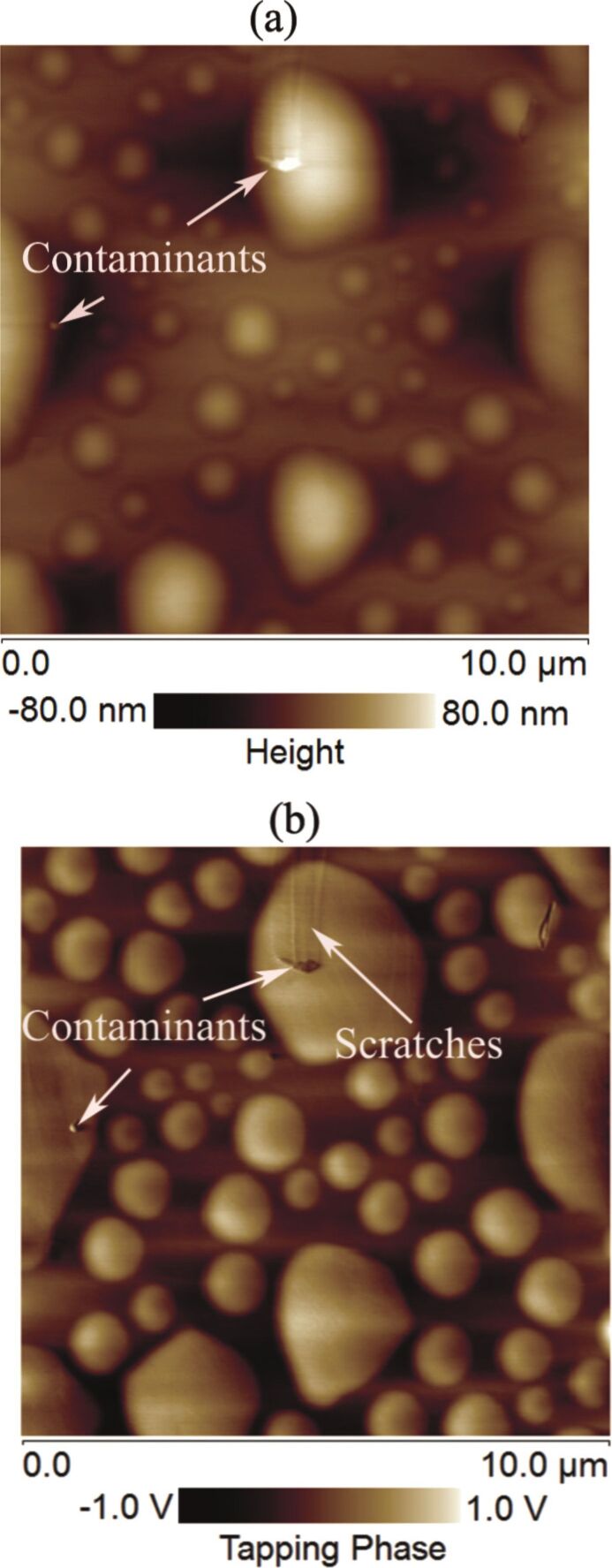
AFM topographic and phase images of PS-coated surfaces obtained in air and water. (a,b) Topographic and phase images, respectively, of the PS-coated surface obtained in DI water. The unknown objects as well as the scratches are clearly visible on the domains. Additional images are given in Supporting Information File 1, Figure S4.

The phase shift is sensitive to the variation in the local surface property [23]. Any change in the surface property can be easily traced through TM-AFM phase contrast analysis. The height images of the spherical domains along with the corresponding phase images are given in Figure 8a,b. The phase contrast in Figure 8b shows a change in the surface property. Furthermore, the height analysis as well as the phase images of these spherical objects showed the presence of contaminants on the top of these domains (Figure 9a,b). Similarly, in some cases, we also observed scratch-like patterns on these spherical domains (Figure 9b).

The scratches and possible contaminants on these domains bring doubt to the possibility that these might be gaseous bubbles, but rather indicate the likelihood of deformed or stretched PS film [26].

### Analysis of the PS film after exposure to water

**Figure 10:**
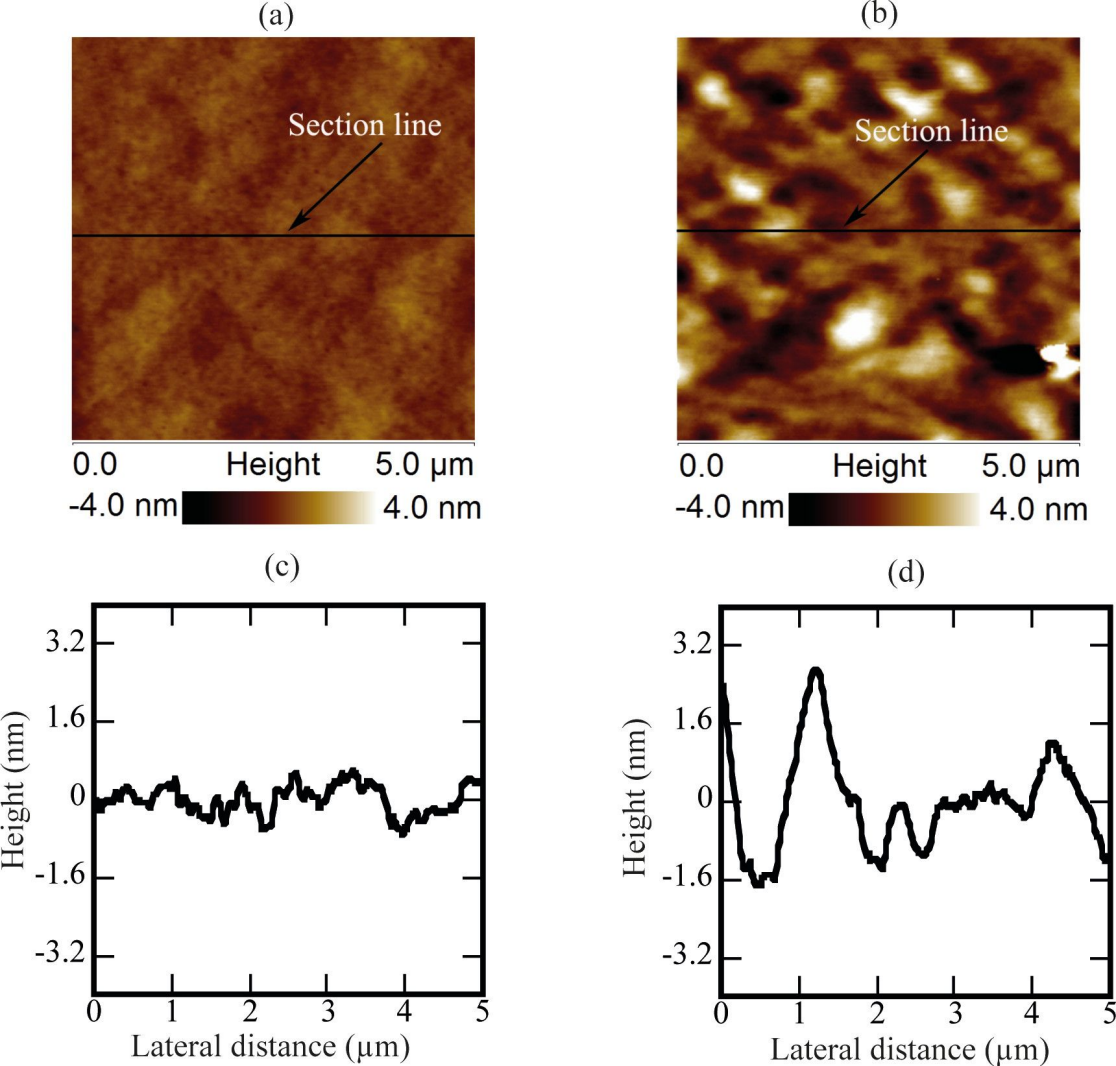
(a,b) AFM images of the PS-coated surface before and after the exposure to water, respectively. (c,d) Height profile analysis of the image of the PS-coated surface before and after the exposure to water. The height profile analysis clearly shows the enhanced features formed on the PS-coated surface. Additional information collected from other surfaces prepared with similar procedures is shown in Supporting Information File 1, Figure S5.

It is proposed that if these objects are not gaseous bubbles, then they are most likely blisters or deformed PS film due to permeation of water through the film [26]. The permeation of water may stretch the PS film and the footprints might be left behind after the removal of water [26]. Therefore, the topography of the PS film before and after the exposure to DI water was also analyzed. The height images, acquired before and after the exposure to water, are shown in Figure 10a–d.

These Figures show that the topography of the PS-coated surface has changed after coming into contact with water. The height section analysis in Figure 10c,d shows that the asperities on the surface exposed to water are comparatively larger than the dry surface.

## Discussion

**Figure 11:**
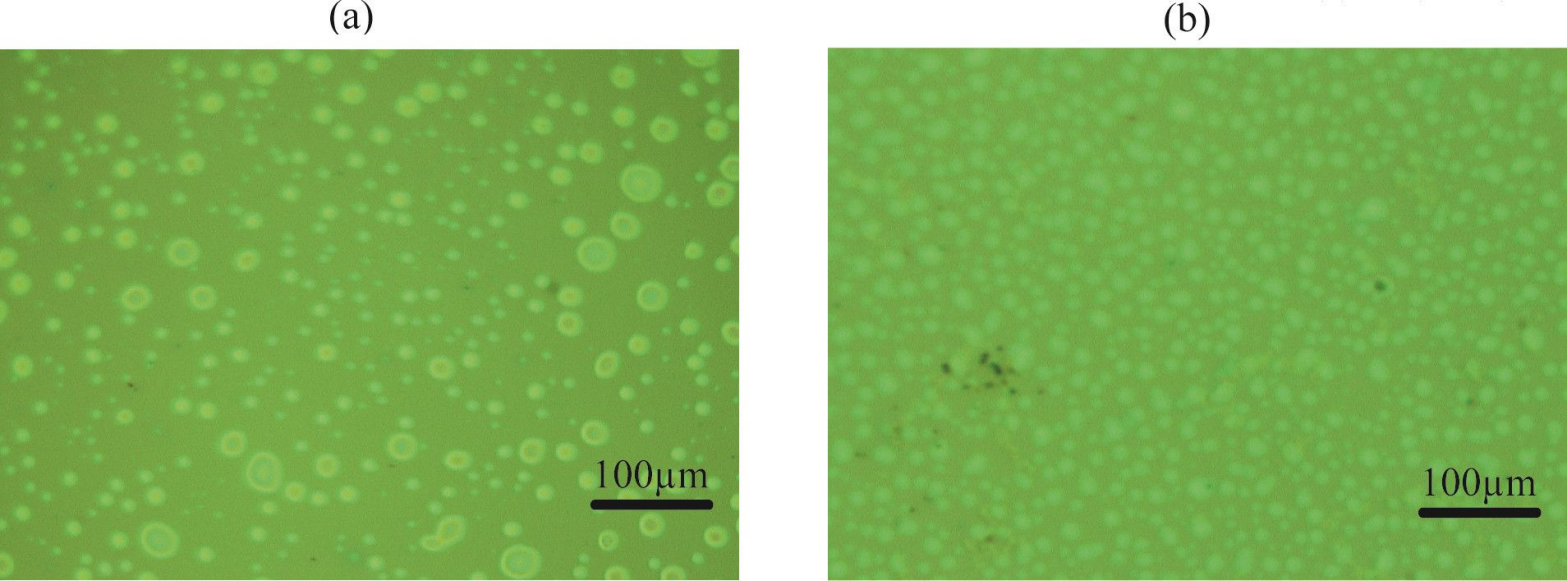
Optical images of blisters formed on a PS-coated surface immersed in (a) DI water and (b) ethanol.

A series of experiments were conducted in order to characterize the spherical or nearly spherical domains that readily nucleate on the PS-coated surface after immersion in water. The focus of this study was to find out whether the micro-/nanobubbles coexist with the spherical or nearly spherical blisters or if these phenomena occur independently. Micrometer-sized spherical objects were found on the PS-coated surfaces. The phase images clearly showed phase contrast at the locations of these objects (Figure 8b). This suggested a change in the local surface property. However, these objects remained insensitive to contact as well as lateral force. Unlike the micro-/nanobubbles, the force measurements did not show long range hydrophobic attraction (Figure 7a). These objects did not show significant movement or deformation during the CM-AFM measurements (Figure 5a,b). Furthermore, contaminants and scratch-like patterns were observed on these spherical objects (Figure 9a,b). To the best of our knowledge, this type of phenomena has not yet been reported for micro-/nanobubbles. In our opinion, contaminants, if present on the surface of the nanobubbles, would not be stable enough to be imaged with CM-AFM or TM-AFM. The presence of the contaminants and scratches highly doubted the idea that these domains could be gaseous bubbles but rather supported the idea that these are the blisters from stretched PS film which appear as spherical micro-/nanobubbles. Additionally, the topographic images obtained before and after contact of water with the PS film showed stretched regions or indents (Figure 10a–d and Supporting Information File 1, Figure S5). A reasonable explanation for the formation of these stretched regions or indents is most likely the penetration of water into the film [26]. As a result, these objects nucleate and leave a footprint as the film dries. Contrary to the optical microscopy study of Berkelaar et al. [26], the proposed blisters were also found upon exposure to ethanol (Figure 11b). Therefore, the hydrophilic silicon surface can further explain the formation of the blisters at the PS–silicon interface. It can be proposed that due to the strong affinity of water towards silicon dioxide, water easily penetrates into the weakly bonded sites of the PS film and the silicon dioxide substrate.

The study of Berkelaar et al. [26] further explained that the smaller water pockets are formed at the defects occurring at the interface of silicon and the PS film. As soon as the osmotic pressure equals the fracture pressure, the PS film detaches from the silicon surface and the blisters begin to form. Their explanation that the water penetrates into the PS–silicon dioxide through defective sites on the PS film is further supported by our study. As was noted, contaminants were present on the blisters as well as on the surface (Figure 9a,b). These defective sites provide a passage for water penetration through the film. This phenomenon leads to the detachment of the PS film from the silicon substrate at the sites that are relatively weakly bonded. Silicon is hydrophilic in nature, and therefore, in the presence of water, the PS film might easily detach. Furthermore, an additional analysis of these domains was also conducted by taking into account the modulus of elasticity of the PS film and the pressure inside the spherical domains. The results are given in Supporting Information File 1, Figure S7. The analysis showed that the surface excess energy approximately ranges from 3.0 to 72.0 nJ/m^2^ for a blister size of 0.5 to 12 µm.

## Conclusion

Spherical domains were found on PS-coated surface. The height, contact angle and line tension analysis suggested a close resemblance of these domains to the previously reported micro-/nanobubbles on the PS-coated surfaces. However the analysis of phase images, force curves and the tip–bubble interaction suggested that these objects are different from the so-called micro-/nanobubbles. The absence of long range hydrophobic attraction force, the formation of scratches and the presence of contaminates on these domains strongly suggested that these are blisters that are formed by the stretching and deformation of the PS film in water. It can be concluded that the presence of contaminates and the defect sites on the PS film provide weak points for the penetration of water through the film. This then causes the detachment of the PS film from the silicon dioxide substrate. The strong affinity of silicon surface towards water further enhances the water permeation and the detachment process of the PS film from the silicon substrate. This can further lead to coalescence of the blisters. Furthermore, blisters can also nucleate in ethanol. The results of the present study support the study of Berkelaar et al. [26] and suggests that the spherical objects, which readily form on the PS-coated surface upon contact with water or ethanol, are most likely blisters formed due to the deformation of the PS film and are not micro-/nanobubbles.

## Supporting Information

Additional information of spherical domains imaged on various surfaces, coalescence at various locations, contaminants and scratches on the domains, surface topographic images before and after exposure to water, additional force curves obtained on different sized spherical domains, and analysis of surface excess energy per unit area.

File 1 Additional experimental information.
