# Supplementary material for: Characterization of spherical domains at the polystyrene thin film–water interface
Source: Beilstein J Nanotechnol. 2016 Apr 20;7:581–90. doi: 10.3762/bjnano.7.51 (PMC4901549; doi:10.3762/bjnano.7.51)
Supplement: File 1 — Additional experimental information. [file Beilstein_J_Nanotechnol-07-581-s001.pdf]

Supporting information

for

Characterization of spherical domains at the polystyrene thin film–water interface

Khurshid Ahmad[1,2], Xuezeng Zhao[1], Yunlu Pan[1]*, and Danish Hussain[3]

[1]Key Laboratory of Micro-Systems and Micro-Structures Manufacturing, Ministry of Education and School of Mechatronics Engineering, Harbin Institute of Technology, Harbin 150001, P.R. China, [2]Department of Mechanical Engineering, Main Campus, University of Engineering and Technology, Peshawar, Pakistan and [3]State Key Laboratory of Robotics and Systems and School of Mechatronics Engineering, Harbin Institute of Technology, Harbin 150001, P.R. China

Email: Yunlu Pan* - yunlupan@hit.edu.cn

* Corresponding author

Additional Experimental Information

AFM images of spherical domains found on PS coated surfaces

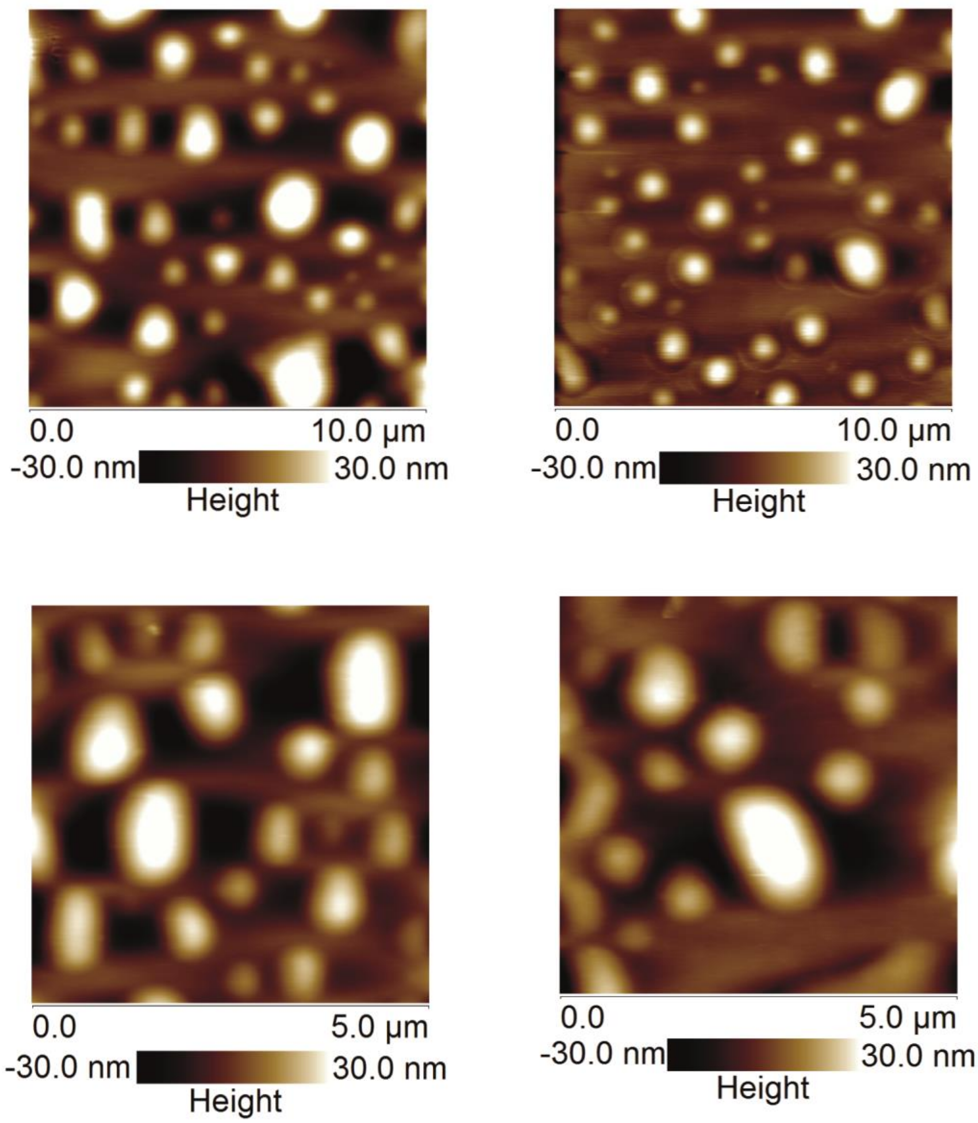

Figure S1: Height images of the spherical domains found on various PS coated surfaces.

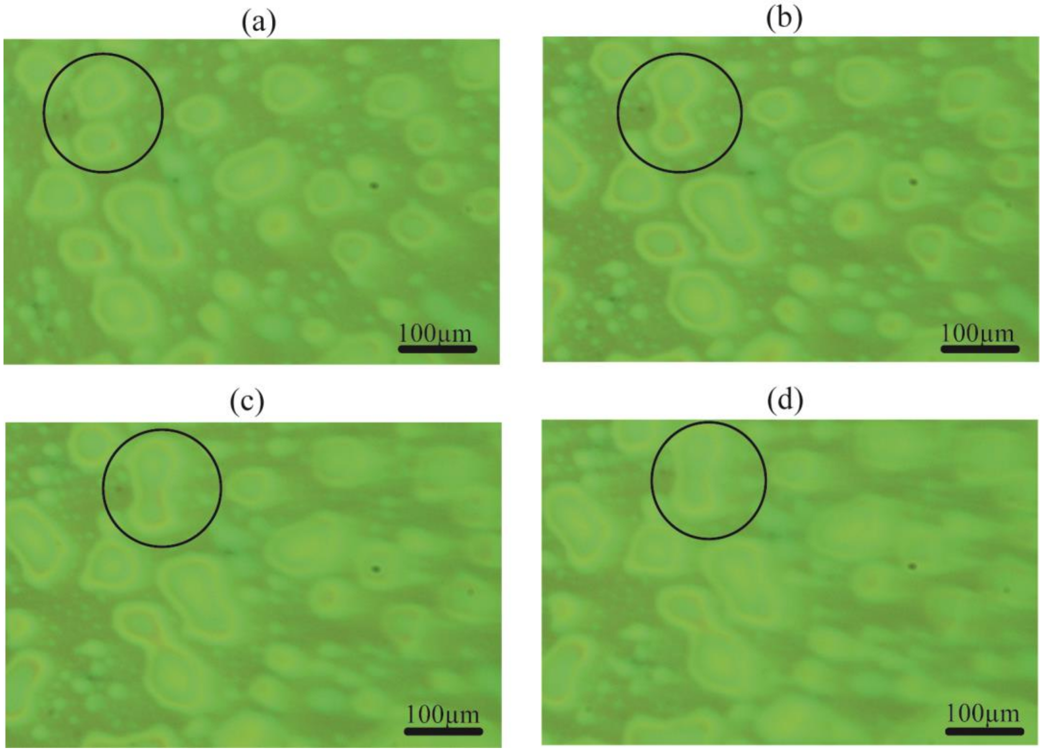

Figure S2: (a-d) Coalescence of two spherical domains is shown here. The encircled areas suggest that the coalescence of two spherical domains may result the formation of an irregular shaped domain.

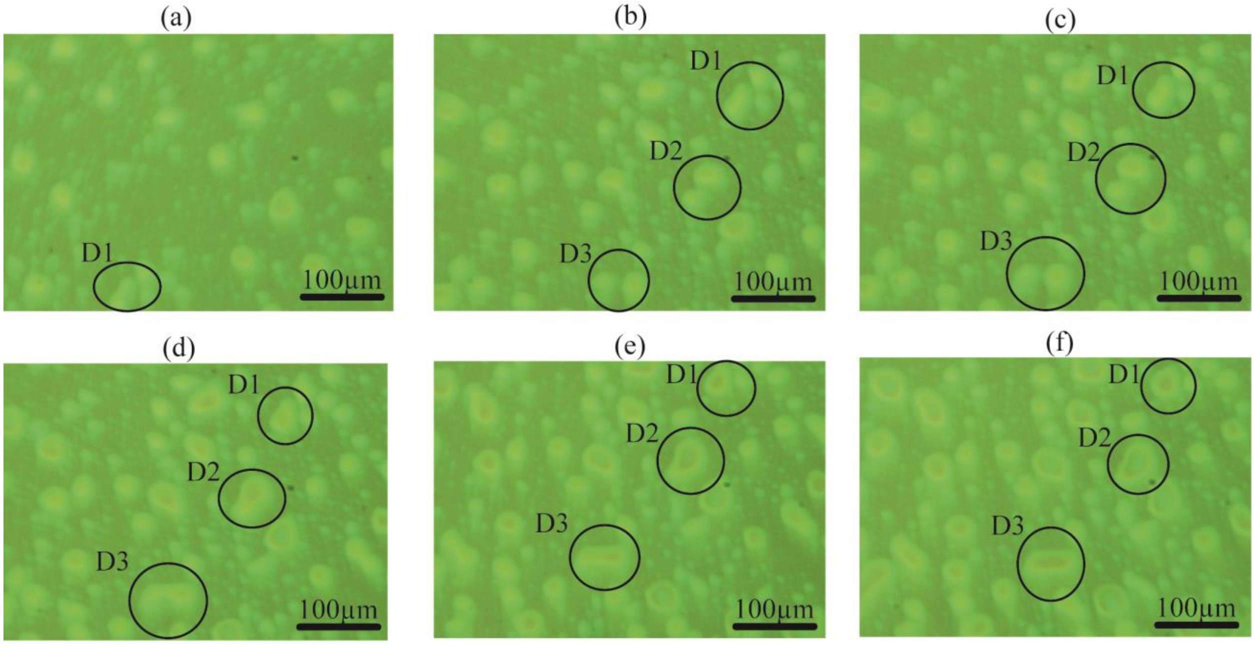

Figure S3: (a–f) Coalescence at three different locations D1, D2 and D3 on PS coated surface is shown here. In this figure it can be noticed that D1 and D2 take a regular spherical or nearly spherical shape but D3 takes an irregular shape as shown in (f). Presence of additional domains in the vicinity of the domains merging together can help in the growth of these domains. Moreover, the presence of additional domains as well as binding of the PS film to the silicon substrate can be the key deciding factors in the shape of these domains resulted from coalescence.

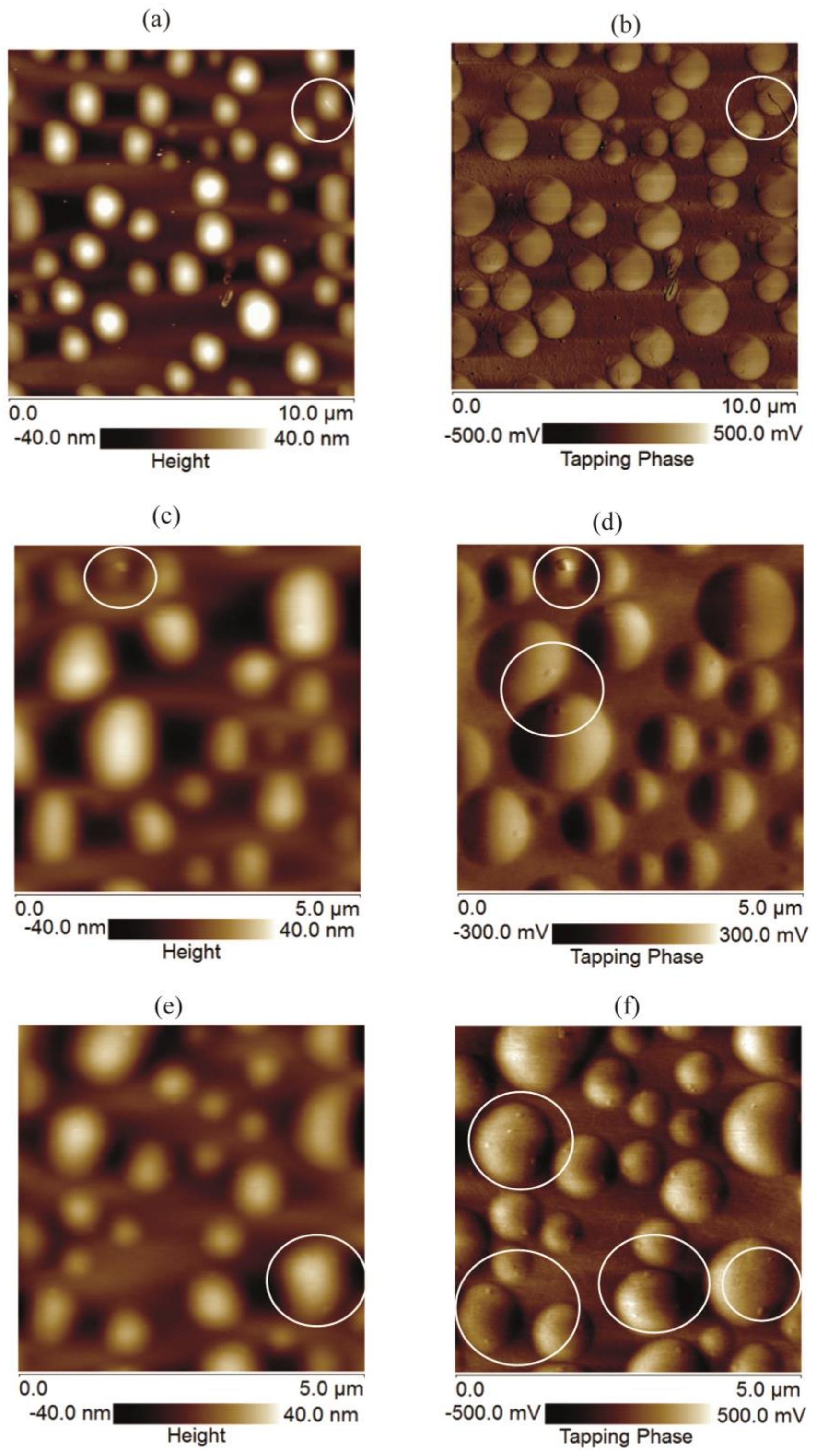

Figure S4: (a–f) height and phase images, respectively, of spherical domains with unknown particles/contaminants shown in the encircled areas. The unknown particles/contaminants are clearly visible in the phase images. The encircled area in (a) shows a scratch on the top of the spherical domain. Similarly, in (d) and (f) the particles can be seen in the areas encircled.

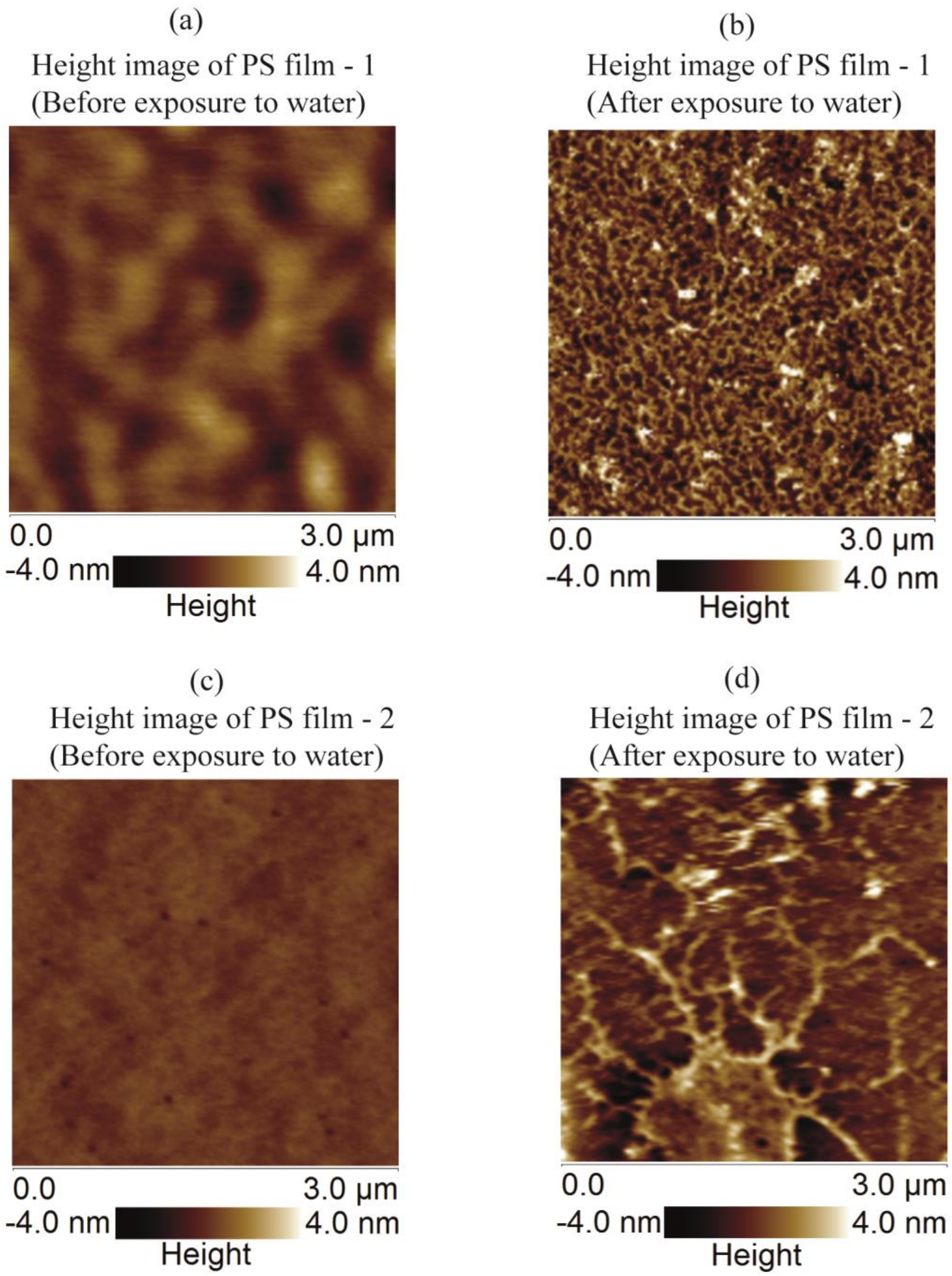

Figure S5: (a) Height image of PS coated surface -1 before exposure to water. (b) Height Image of PS coated surface -1, after exposure to water. (c) Height image of PS coated surface -2, before exposure to water. (d) Height image of PS coated surface -2, after exposure to water. An obvious difference in the topographic features is visible.

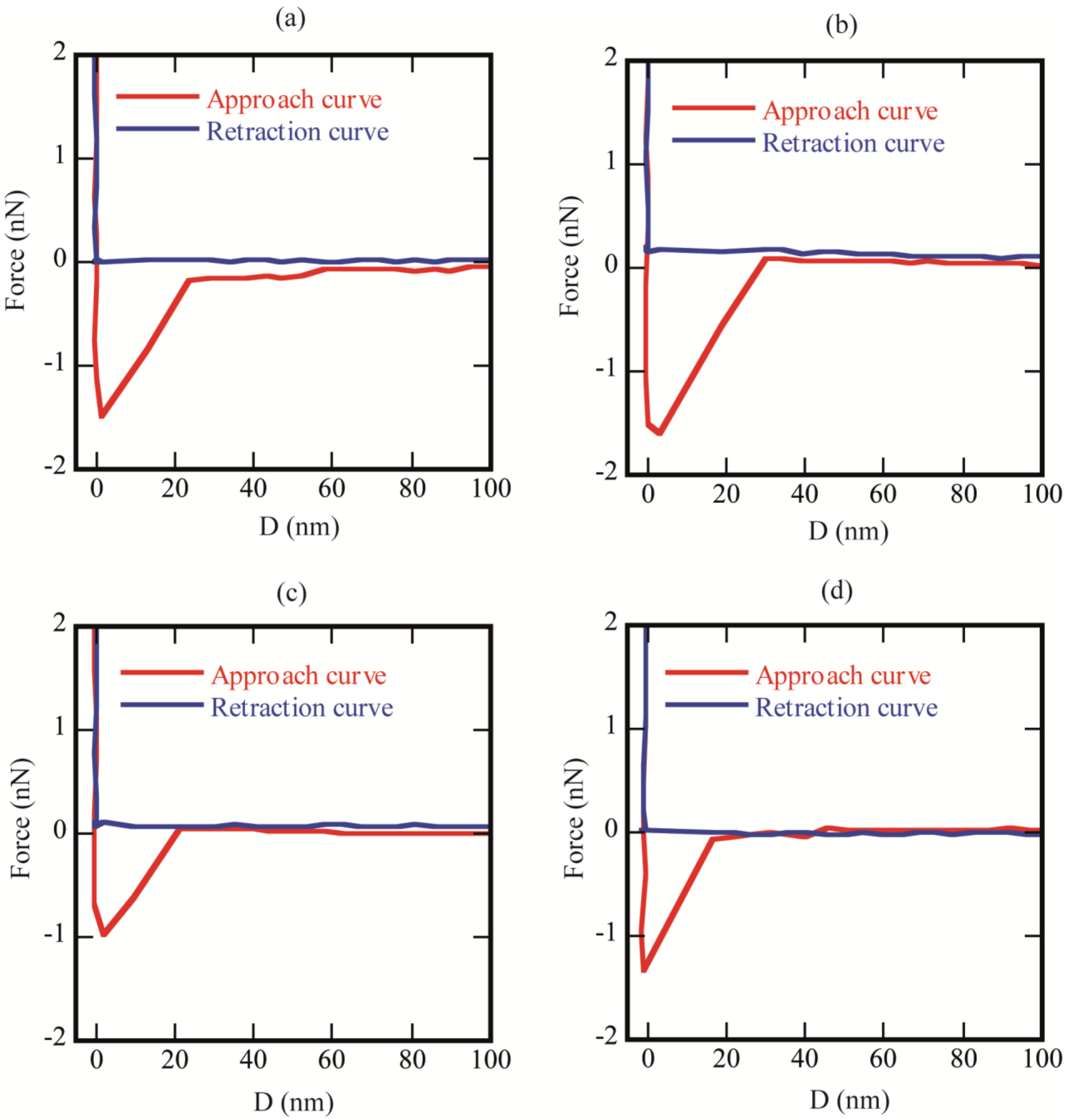

Figure S6: (a) Approach and retraction curve of PS surface. (b) Approach and retraction on spherical domain with Radius/later size, R = 1.3 µm, Height, H = 22 nm. (c) Approach and retraction on spherical domain with Radius/later size, R = 0.9 µm, Height, H = 16 nm. (d) Approach and retraction on spherical domain with Radius/later size, R = 0.6 µm, Height, H = 6 nm.

Analysis of surface energy

The analysis of the excess energy per unit area was also conducted. The modulus of elasticity of polystyrene (PS) film and the pressure inside the blister was taken into account. The critical stress (σ_c) can be given by equation 1[1, 2].

$$\sigma_c = \sqrt{\frac{2E\gamma}{\pi R_b}} \qquad (1)$$

Where, E is the modulus of elasticity of the PS film, γ is the excess surface energy per unit area. And R_b is the radius/lateral size of the blister. At equilibrium the vapour pressure (P_i) at room temperature will be equal to the σ_c therefore, equation 1 will take the form:

$$\gamma = \frac{\pi P_i^2 R_b}{2E} \qquad (2)$$

where $E = 2.6\ GPa$ [3], $P_i = 3.169 kPa$ [4] and R_b was obtained from the AFM images. The R_b ranged from 0.5 µm to 12 µm. Using this data, the surface excess energy was calculated and is plotted against the R_b as shown in Figure S7.

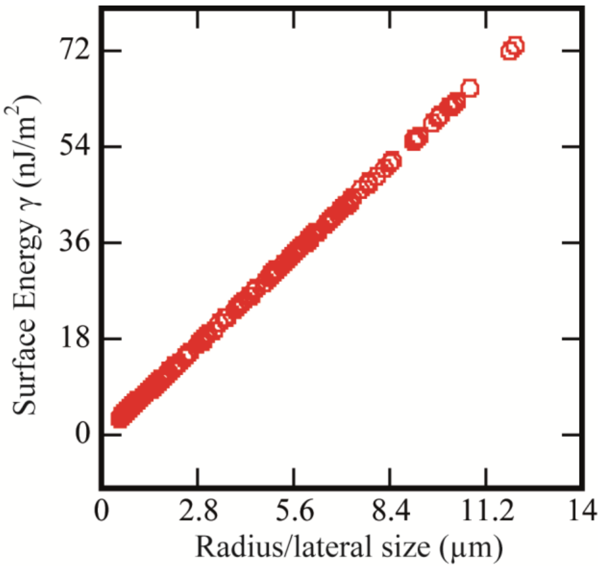

Figure S7: Variation of surface excess energy with respect to the radius/lateral size of the blister.

References

1. Berkelaar, R. P.; Bampoulis, P.; Dietrich, E.; Jansen, H. P.; Zhang, X. H.; Kooij, E. S.; Lohse, D.; Zandvliet, H. J. W. *Langmuir* **2015,** *31* (3), 1017-1025.
2. Griffith, A. A. *Philosophical transactions of the royal society of london. Series A, containing papers of a mathematical or physical character* **1921,** *221*, 163-198.
3. Du, B.; Tsui, O. K. C.; Zhang, Q.; He, T. *Langmuir* **2001,** *17* (11), 3286-3291.
4. Lide, D. R. *CRC Handbook of Chemistry and Physics*, CRC Press, Boca Raton, FL, **2005**.
